# Critical factors that affect the functioning of a research and evaluation capacity building partnership: A causal loop diagram

**DOI:** 10.1371/journal.pone.0262125

**Published:** 2022-01-13

**Authors:** Rochelle Tobin, Gemma Crawford, Jonathan Hallett, Bruce Richard Maycock, Roanna Lobo

**Affiliations:** 1 Collaboration for Evidence, Research and Impact in Public Health, School of Population Health, Curtin University, Perth, Western Australia, Australia; 2 European Centre for Environment and Human Health, College of Medicine and Health, University of Exeter, Devon, South West England, United Kingdom; Universiti Teknologi Malaysia - Main Campus Skudai: Universiti Teknologi Malaysia, MALAYSIA

## Abstract

**Introduction:**

Public health policy and practice is strengthened by the application of quality evidence to decision making. However, there is limited understanding of how initiatives that support the generation and use of evidence in public health are operationalised. This study examines factors that support the internal functioning of a partnership, the Western Australian Sexual Health and Blood-borne Virus Applied Research and Evaluation Network (SiREN). SiREN aims to build research and evaluation capacity and increase evidence-informed decision making in a public health context.

**Methods:**

This study was informed by systems concepts. It developed a causal loop diagram, a type of qualitative system model that illustrated the factors that influence the internal operation of SiREN. The causal loop diagram was developed through an iterative and participatory process with SiREN staff and management (n = 9) via in-depth semi-structured interviews (n = 4), workshops (n = 2), and meetings (n = 6).

**Results:**

Findings identified critical factors that affected the functioning of SiREN. Central to SiREN’s ability to meet its aims was its capacity to adapt within a dynamic system. Adaptation was facilitated by the flow of knowledge between SiREN and system stakeholders and the expertise of the team. SiREN demonstrated credibility and capability, supporting development of new, and strengthening existing, partnerships. This improved SiREN’s ability to be awarded new funding and enhanced its sustainability and growth. SiREN actively balanced divergent stakeholder interests to increase sustainability.

**Conclusion:**

The collaborative development of the diagram facilitated a shared understanding of SiREN. Adaptability was central to SiREN achieving its aims. Monitoring the ability of public health programs to adapt to the needs of the systems in which they work is important to evaluate effectiveness. The detailed analysis of the structure of SiREN and how this affects its operation provide practical insights for those interested in establishing a similar project.

## Introduction

Improvement in public health policy and practice is supported by the capacity to generate and apply evidence to decision-making [1–4]. Consequently, initiatives to increase evidence-informed decision-making should consider the intersecting roles of knowledge translation and research and evaluation capacity building. Knowledge translation focuses on the creation of relevant and useful evidence and facilitating its exchange and application [4]. Research and evaluation capacity is concerned with strengthening research and evaluation abilities and using findings to inform decision-making [5–7]. This study examines the mechanisms underpinning the function of unique partnership that uses both knowledge translation and capacity building strategies to increase evidence-informed decision making within a public health context.

A substantial body of literature explores different knowledge translation and research and evaluation capacity-building strategies. These include tailored support, training, resources and tools (e.g. knowledge platforms), leadership support, partnerships, and increasing access to funding [2, 8–11]. Of these strategies, partnerships that increase the proximity of researchers and knowledge users support the generation and application of practical evidence to decision making [2, 12–14]. These partnerships enable researchers and knowledge users to build relationships based on reciprocity, trust, and respect, facilitating mutual learning [14]. There is growing interest in partnership-based and multi-strategic approaches that work across individual, organisational and system levels [2, 8, 9, 14]. Despite this interest, there is little empirical evidence of how partnership approaches may be designed and operationalised [2, 8, 11, 13].

The partnership approach examined in this paper is the Western Australian Sexual Health and Blood-borne Virus Applied Research and Evaluation Network (SiREN). SiREN is a public health intervention that works within a system of government, research and non-government organisations that prevent and manage sexual health and blood-borne virus (SHBBV) issues (the system). SiREN uses a multi-strategic, context-specific approach [2, 10, 15]. Impacts achieved previously by SiREN include the development of research and evaluation knowledge, skills, and confidence; increased research and evaluation funding and the establishment of networks [16].

SiREN is an example of a complex intervention. Willis et al. [17] described a complex intervention as one designed to meet the needs of the context in which it operates, incorporating a range of strategies that target different levels of a system, and with strategies that operate independently and interdependently. Given the complexity of SiREN, a systems approach was selected to investigate its functioning. A systems approach draws on a variety of concepts and methods to explore complex phenomena [18]. It defines the boundaries of the system or situation of interest, explores its structure, and identifies how its elements interact to bring about change [19–21]. A recent review highlighted a need for evaluators to test systems methods and share their findings to guide others interested in applying systems approaches to evaluation [19].

A systems approach has not been used to explore a research and evaluation capacity-building project to the authors’ knowledge. One approach that has been used to examine the complexities of such projects is a realist approach. This method has been used to explore research capacity building [2] and knowledge mobilisation [22] projects. A realist approach is well suited to exploring complex programs as it seeks to understand what works and why in different contexts [2]. It is similar to a systems approach in two main ways. Firstly, they are based on the assumption that programs can be viewed as complex events occurring within complex systems [22]. Secondly, they pay attention to context and causal relationships [22]. Studies have concluded that a realist approach provided a nuanced understanding of the processes by which capacity building and knowledge translation projects contributed to change [2, 22]. These findings indicate complexity sensitive methods will generate useful insight into the operation of SiREN.

System modelling was used to understand SiREN, specifically causal loop diagramming. This qualitative method visually depicts a bounded system or situation and how its components interact to generate change [23, 24]. The system variables are named and joined with arrows to illustrate their relationships [25]. The relationships between variables can form feedback loops. These are cyclical processes of change that either amplify an effect (a reinforcing loop leading to increases) or inhibit it (balancing loop leading to stability) [23]. For example, there will be multiple variables that influence the ability of a public health project to achieve its aims. These variables include those that increase effectiveness (e.g. access to additional funding) and those that reduce effectiveness (e.g. poor stakeholder engagement). Furthermore, these variables will interact, making the implementation of SiREN complex [26].

Several studies have used causal loop diagrams to explore the implementation or operation of a program or strategy [26–30]. Fredericks et al. [28] concluded that the development of a causal loop diagram provided insight into factors that affect variability in program implementation, highlighted competing goals within the system and identified critical feedback processes and unintended consequences. Brown et al. [30] described how creating the diagram with stakeholders deepened their understanding of their programs and led to the development of useful indicators for ongoing monitoring and evaluation. Despite its relatively limited application in this context, those who have utilised causal loop diagrams concluded that they gained practical insight into how the organisational dynamics of a project affected its success [26, 27, 29].

### Aims

This research aimed to examine the organisational dynamics that influence the functioning of a research and evaluation capacity building partnership (SiREN). This paper describes SiREN, presents and discusses a causal loop diagram that illustrates factors that affect its operation, and provides insight for others interested in building capacity to engage in research, evaluation or evidence-informed practice in a public health context. This research forms part of a larger study that explores the external processes and outcomes of SiREN [31].

## Methods

A causal loop diagram was developed to illustrate the factors (e.g. governance, funding, staffing) that influence the internal functioning of SiREN. The causal loop diagram was developed in an interactive process with SiREN staff and management (n = 9), including three research team members via in-depth semi-structured interviews (n = 4), workshops (n = 2), and meetings (n = 6). The study was approved by the Curtin University Human Research Ethics Committee (approval number: HRE2017-0090).

### SiREN

The concept for SiREN began in 2009, formulated by a group of researchers, policymakers and service providers seeking a way to support the generation of evidence to inform the response to local SHBBV issues. SiREN was formally funded and established in 2012 to strengthen evidence-informed practice through building research and evaluation capacity and promoting opportunities for collaboration between researchers, policymakers and service providers working to address SHBBV issues. SiREN is led by a management team (described below) and a steering group composed of stakeholders from policy, practice and research settings. Its strategies include: developing partnerships for research and evaluation; co-creating research and evaluation evidence; providing tailored program planning, research and evaluation support; delivering training and resources; and sharing knowledge through a network of over 450 members. A detailed description of SiRENis provided elsewhere [16, 31].

### Participants

Participants included SiREN staff and management team members (n = 9). SiREN staff were research officers, project officers and project coordinators working on various research, evaluation and knowledge translation related projects. The management team comprised (n = 5) University staff, involved in the operational and strategic management of SiREN. Their roles within the University were research, teaching and project management related.

### Theoretical framework

This research used a systems approach to investigate factors and interactions that affect the operation of SiREN. Systems approaches explore structures, relationships and patterns [18, 21, 32]. They directly contrast reductionist approaches that break down programs into their component parts and draw direct links between program strategies and effects [32]. Instead, systems thinking acknowledges that understanding a phenomenon requires viewing it holistically, with attention paid to how its parts interact to affect change [32]. Applying systems thinking concepts and methods can create a comprehensive shared understanding of programs that can be used to inform decision-making regarding implementation [33].

In this study, three fundamental tenets of a systems approach were applied to design the inquiry; boundaries, perspectives and relationships. Establishing boundaries sets the scope for what is to be explored, enabling clear lines to be drawn between what is pertinent and what is not [18, 34]. Boundaries are defined variously, including through organisational, geographical, or social means [35]. For analysis, SiREN is the bounded situation, factors outside of SiREN’s control were not included. Taking a narrower focus supported a deeper exploration of SiREN and provided greater insight into how a model like SiREN may be implemented in another context [27]. The concept of perspectives acknowledges that each system stakeholder may hold a different view of reality. Modelling approaches that combine different perspectives can encourage shared learning and develop a more comprehensive view of the situation [18, 36]. Given the focus on the internal operation of SiREN, perspectives were sought from those working within SiREN. The concept of relationships demonstrate how variables within the situation of interest interact and influence each other to achieve an aim [35]. Because of this, relationships can be more important than the variables themselves in understanding the behaviour of the system [32]. In this study, relationships were elucidated through the development of a causal loop diagram.

### Research team and reflexivity

During the time this study was undertaken four of the authors (RT, RL, JH and GC) had worked with SiREN. RL was the SiREN manager and RL, JH and GC were members of the SiREN management team. RT had previously worked with SiREN as a project officer. BM has not previously worked for SiREN. BM is a senior and experienced public health academic with many decades of experience in research and practice. All research team members had experience working with SHBBV health issues, public health, qualitative research, and evaluation. Several members had experience in public health practice and policy. Two members had previously worked for a non-government organisation within the system.

As most of the research team (RT, RL, GC, JH) were past or present SiREN staff or management team members we considered this study to be insider research. Insider research has its unique strengths and challenges [37, 38]. The strengths associated with being an insider included a strong understanding of the subject matter and existing relationships between researchers and participants that can support data collection and analysis [38]. This was evident in interviews where the familiarity between researcher and participants supported the development of a safe space to share information. It was also apparent in workshops where the rapport between participants enabled them to easily build on each other’s ideas and question opinions non-confrontationally. A challenge of insider research is a loss of objectivity, assuming shared understanding or overlooking data that appear commonplace to an insider [39–41]. To overcome this, during data collection participants were asked to explain situations where knowledge was assumed and during data analysis RT was mindful not to dismiss data that seemed obvious as an insider. BM, who was not directly involved with SiREN, reviewed the data and results to increase trustworthiness and consistency with analysis [37]. Participants were given an opportunity to validate the final causal loop diagram in a workshop and field notes were developed after interviews and workshops, which supported rich descriptions of context and enabled the researcher to reflect on and identify bias [42].

### The development of the causal loop diagram

This study used a three-stage process to develop and validate a causal loop diagram, described in the following section. This process included collecting data, developing a draft diagram, and validating the diagram with study participants. Data analysis processes were informed by those generated by Kim and Anderson [43] and the diagram was verified with stakeholders using similar processes articulated in comparable studies [28, 44, 45]. The Consolidated Criteria for Reporting Qualitative Research (COREQ) checklist was used to guide reporting [46].

#### Step one: Data collection to inform the development of the causal loop diagram

Data were collected through a workshop with the Project management team and interviews with SiREN staff to inform the initial causal loop diagram development. RT undertook all data collection and analysis.

*Management team workshop*. A workshop was held with the management team (n = 5). The aims of the workshop were to determine: what factors affect the functioning of SiREN; how and why SiREN has changed since it was established; and where SiREN needs to go in the future. A workshop was considered the most appropriate data collection method as it would enable the collective development of opinions and ideas [47] In the workshop, the facilitator (RT) posed questions to the group that directly reflected the workshop aims. Participants discussed their responses as a group, questioning and building on points raised by other participants. The workshop ran for 50 minutes and was audio-recorded and transcribed verbatim by a transcription service and checked by RT.

*Staff interviews*. SiREN staff members (n = 4) who had been employed in the previous 12 months were contacted via email and invited to participate in semi-structured, qualitative interviews. All staff agreed to participate. Participants held an undergraduate degree or higher and worked with SiREN between one and eight years. Their roles within SiREN included senior research officer, project officer, project coordinator, and project administrator. Three were current staff members; one had ceased employment six months previously.

Interviews were selected as the most appropriate data collection method to address possible power dynamics between staff and management team members that may have arisen in a group environment. It was anticipated that staff would feel more comfortable sharing information individually rather than in group conversation. Using interviews provided a confidential environment for staff to share their experiences and views and have them incorporated into the diagram before it was presented to the group. As the research team is involved with SiREN’s management, RT provided assurances to participants that participation or declining participation would not affect their employment or relationships with SiREN. RT also described how data would be managed to ensure that staff were aware that the management team would not have access to identifiable data [37]. The authors note that given the team’s small size, it may be possible for participants to identify each other’s input. To reduce this likelihood, no quotes were shared that contained information that may reveal the participant’s identity.

Interviews were undertaken using Microsoft Teams video conferencing software [48] due to social distancing measures taken during the COVID-19 pandemic. As the researcher knew the participants from previously working together, rapport was easy to establish online. The interviews explored the same areas as the workshop with the management team, with additional questions relating to the staff members’ role within SiREN. The research team developed the interview schedule (see S1). Interviews ranged in duration from 30 to 80 minutes and were audio recorded.

#### Step two: The development of the causal loop diagram

To develop the causal loop diagram, the workshop and interviews were transcribed verbatim by a transcription service and the transcripts were checked by a researcher (RT) for accuracy. Transcripts were not returned to workshop or interview participants for comment or correction as they were given an opportunity to validate the findings in a subsequent workshop to refine the causal loop diagram. Transcripts from the workshop and interviews and field notes were entered into QSR NVivo 11 data management software [49]. Using the grounded theory-informed approach recommended by Kim and Anderson [43], data were open coded to identify variables that affected the operation of SiREN. Data were analysed using manifest qualitative content analysis, thus staying close to what participants actually said [50]. The process of coding was iterative and involved reviewing the data and refining and grouping codes into categories until no new variables emerged and several dominant categories were identified. This resulted in further refining and narrowing the boundaries of the enquiry. Initial data analysis explored external and internal factors that affect the operation of SiREN, the history and future directions of SiREN, and its perceived value. As described in the literature [51], attempting to include all of these aspects of SiREN in one diagram would make it too complicated. Therefore, the diagram focused only on internal factors that affected the operation of SiREN. Axial coding identified causal relationships between variables e.g., the variable ‘*shared vision*’ had a positive relationship with the variable ’*cooperation between management team members*’. These relationships formed the arrows in the causal loop diagram.

During the coding process a table was developed in Microsoft Excel (Version 2105) that included variables, relationships and supporting data (see. Table 1). Adapted from Kim and Anderson [43], table’s purpose was to create an audit trail of transparent and traceable links between data and the diagram, building confidence in the diagram’s reliability. Developing the table facilitated the identification of inconsistencies or previously unidentified relationships and improved the diagram’s accuracy.

**Table 1:** Coding table example.

Variable	Effect variable	Relationship type	Supporting data and source
Knowledge of the system	Adaptability	Positive	*“We have more insight into around what is happening in the sector because of those partnerships and so we are able to respond quicker*…*”* Source: Interviews, P6

#### Step three: Validation of the causal loop diagram

Validation involved refining the draft causal loop diagram through a series of consensus-building activities. The research team held three one-hour meetings (RT, RL, GC, JH). At each meeting, RT presented the latest version of the diagram. The group discussed the phrasing and meaning of variables, the nature of the relationships and identified missing variables or relationships. In addition, the research team identified themes: points within the diagram where related variables and relationships converged. These themes were deemed as central to the operation of SiREN. RT then refined the diagram based on this feedback. This process continued until no new variables or relationships emerged.

*Combined management team and staff workshop*. Once the research team agreed that the diagram sufficiently represented SiREN, RT held a two-hour workshop with SiREN staff and management team members. To facilitate understanding, the diagram was separated into three smaller diagrams. RT allocated participants into small groups, which were rotated to discuss the diagrams. Participants were asked to examine the diagram variables and relationships and describe any missing variables or relationships. Working in smaller groups ensured that each participant had the opportunity to contribute, a process used in a similar study [36]. The group then reconvened to share their insights and review the whole diagram. Separate meetings were held with one management team member who could not attend the workshop and with two research team members (GC, JH). At this point, there was consensus that the diagram was an accurate depiction of SiREN and data collection ceased.

## Results

**Fig 1:**
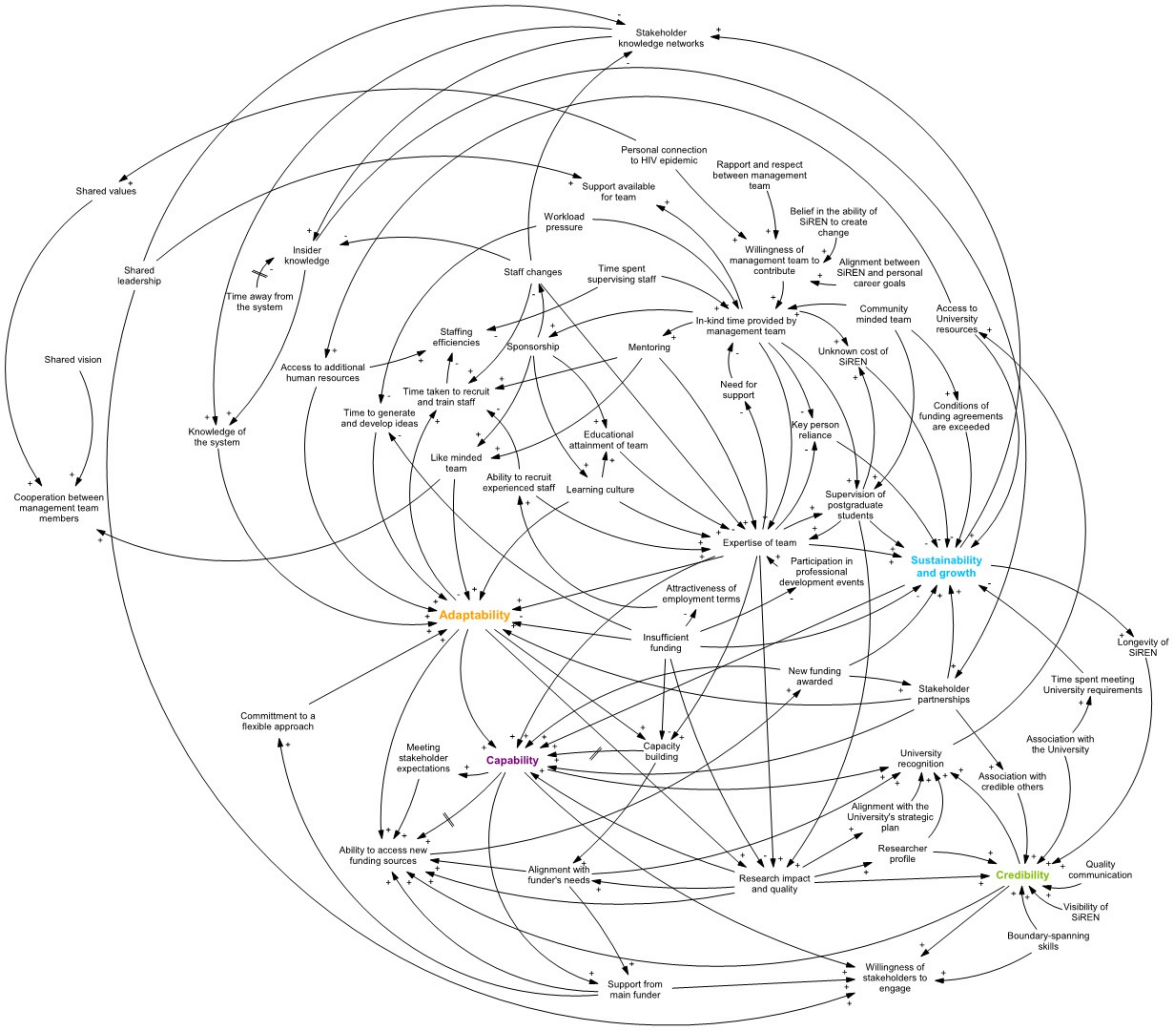
Causal loop diagram illustrating the operation of SiREN.

Fig 1 presents the causal loop diagram illustrating factors that influence the functioning of SiREN. To read the diagram, start with a variable of interest and follow its relationships through to other variables. To support readers’ understanding of the diagram, variables have been italicised in the narrative description, and the S1 Table in S1 File presents glossary of the diagram variables.

The diagram illustrates four main themes depicted in coloured text: adaptability, capability, credibility, and sustainability and growth. These themes were identified as variables that were fundamental to the success of SiREN and were used to frame the discussion of the results. The meaning of the themes and their relationships to diagram variables are presented in Table 2.

**Table 2:** Definition and linkages of causal loop diagram themes.

Themes	Definition	Influencing variables^1^ (relationship type +/-)	Effect variables^2^ (relationship type +/-)
Adaptability	How SiREN learns from the system and adjusts its processes and activities to respond (e.g. changes in epidemiology).	Access to additional human resources (+) Commitment to a flexible approach (+) Expertise of team (+) Insufficient funding (-) Knowledge of the system (+) Learning culture (+) Like-minded team (-) Stakeholder partnerships (+) Time taken to recruit and train staff (+)	Ability to access new funding sources (+) Capability (+) Capacity building (+) Research impact and quality (+) Time to generate and develop ideas (+)
Capability	The extent to which SiREN can undertake its activities and achieve its aims.	Adaptability (+) Capacity building (+) Expertise of the team (+) New funding awarded (+) Research impact and quality (+) Stakeholder partnerships (+) Sustainability and growth (+)	Ability to access new funding sources (+) Meeting stakeholder expectations (+) Support from main funder (+) University recognition (+) Willingness of stakeholders to engage (+)
Credibility	The extent to which SiREN is a trusted and believable source of knowledge.	Association with credible others (+) Association with University (+) Boundary-spanning skills (+) Longevity of SiREN (+) Quality communication (+) Research impact and quality (+) Researcher profile (+) Visibility of SiREN (+)	Ability to access new funding sources (+) University recognition (+) Willingness of stakeholders to engage (+)
Sustainability and growth	The ability of SiREN to acquire and utilise resources to grow and maintain its activities and achieve its aims. Resources include financial, human resources and partnerships.	Access to University resources (+) Conditions of funding agreements are exceeded (-) Expertise of team (+) Insufficient funding (-) Key person reliance (-) New funding awarded (+) Stakeholder partnerships (+) Supervision of postgraduate students (+) Unknown cost of SiREN (-)	Capability (+) Longevity of SiREN (+) Time spent meeting University requirements (-)

^1^Variables that affect the theme. Direction of the relationship is from the variable to the theme.

^2^Variables that the theme affects. Direction of the relationship is from the theme outwards.

### Adaptability

The *adaptability* of SiREN was vital to achieving its aims. Participants described SiREN as dynamic and explained that it has evolved in response to changes in the system in which it is nested. Participants reported that *adaptability* was enhanced by SiREN’s *commitment to a flexible approach*. This was demonstrated by the team continually seeking to improve its structure and activities. The team were curious about trying new approaches and working in different areas. With support from its main funder, SiREN maintained a flexible approach, enabling modification of its activities to respond to changes within the system:

*“I think that the flexible governance of SiREN*…*allowed us to do that*…*rather than being too rigid*. *We can kind of go*, *well*, *we*’*re going to have to try all this and we*’*re going to have to be okay with that*, *rather than saying*, *well*, *it has to be delivered in this particular way*.*”*

*(P2)*

*Stakeholder partnerships* and *stakeholder knowledge networks* enhanced SiREN’s *knowledge of the system* by facilitating knowledge exchange. *Knowledge of the system* was gained through formal processes such as project steering group meetings and stakeholder needs assessments and informal processes such as networking events. Participants understood the critical role *knowledge of the system* plays in supporting *adaptability* as it enabled them to see what was required. Participants observed that the free flow of knowledge between stakeholders and SiREN necessitated the presence of trusting relationships. As the system constantly fluctuates, participants had limited capacity to keep abreast of all relevant changes, consequently acknowledging their understanding of the system was incomplete. However, *stakeholder partnerships* boosted *adaptability*, by enabling an informed and rapid response to emerging issues:

*“We have more insight into around what is happening in the sector because of those partnerships and so we are able to respond quicker to things even when it is not yet in the epidemiology*, *but we know that is an issue because we are hearing it from organisations*. *So*, *in many ways those connections to these organisations have allowed us to kind of respond quicker to some of their needs*.*”*

*(P6)*

**Fig 2:**
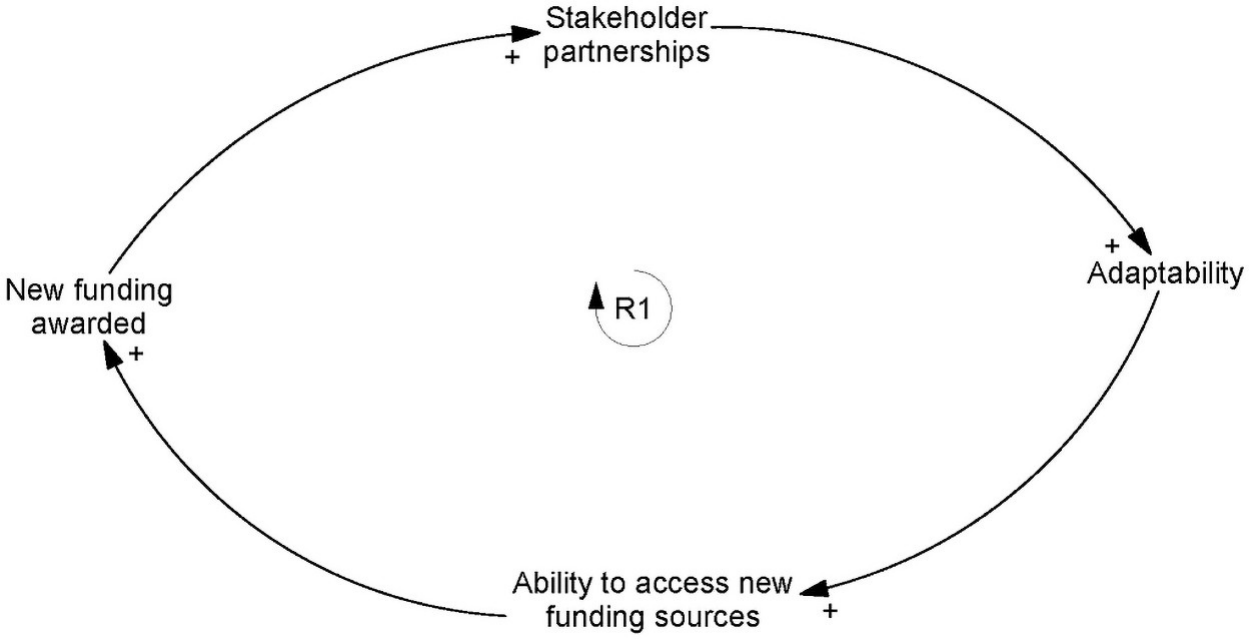
Reinforcing loop one: Adaptability, funding and partnerships.

*Adaptability* improved SiREN’s *ability to access new funding sources* as new opportunities were identified. The acquisition of new funding sources, in turn, increased the number and strength of *stakeholder partnerships*, and in turn, the capacity of SiREN to adapt (Fig 2). Temporality was a consideration with the time taken to build these partnerships explained by a participant:

*“The relationship building took two to four years to be of any value*, *to really start to be able to apply for grants and to do significant work together*.*”*

*(P5)*

The *insider knowledge* held by many management team members was highlighted by participants as another critical source of knowledge. This *insider knowledge* came from the management team’s history of working with and within stakeholder organisations. From these experiences, they had relationships to draw on to support the work of SiREN. As pseudo insiders they also understood how the system worked, who the decision makers were, and appropriate ways to respond to the system’s needs. This was reported by a participant:

*“I think there*’*s that connection with the sector and also them knowing you makes it (SiREN) work*… *there*’*s more of a reputation*.*”*

*(P3)*

Participants explained how the *insider knowledge* held by the management team supported SiREN as it became established. As University employees, many management team members have now spent several years or more with limited contact with stakeholders, which has reduced their level of *insider knowledge*. SiREN staff now hold some of this knowledge, developing their understanding over several years working with SiREN and its stakeholders. A participant observed that these relationships were essential in the early days of SiREN, but over time it developed its own identity:

*“But some of that insider knowledge that we did have*…*a lot of that*’*s evaporated*… *SiREN has picked up the mantle of its own relationship to the NGO (non-government organisation) sector*, *so it*’*s not reliant on our previous or historic connections*. *SiREN now has its own reputation*.*”*

*(P1)*

SiREN’s *shared leadership* structure (shared between the management team and steering group) supported inclusive decision-making on how it will achieve its aims. Participants noted that sharing leadership increased the *willingness of stakeholders to engage* as they become familiar with SiREN. Historically, *shared leadership* played an essential role in establishing the *shared vision* of what SiREN aimed to achieve as key stakeholders had a say in setting SiREN’s aims. The presence of a *shared vision* strengthened the *cooperation between management team members*:

*“You have the shared vision around what we*’*re actually trying to achieve*… *everyone*’*s kind of invested in achieving that*, *then the uniqueness of the personalities come together*.*”*

*(P1)*

The presence of *shared values* increased *cooperation between members of the management team*. Their *shared values* were formed through personal and professional experience predating the inception of SiREN. While the management team’s *shared values* were implicit, they recognised their role in guiding decision-making and acknowledged that their cooperative nature would have been compromised without them.

The management team reflected a level of like-mindedness due to similar work experiences and values. However, being a *like-minded team* could reduce *adaptability* by limiting innovation. The team reported that like-mindedness could negatively affect the creation of new ideas and ability to challenge each other’s ways of thinking, requirements for innovation:

*"*…*having like-minded people can also be a negative*. *Those habitual practices*, *you know*, *having expectations that are not*, *kind of*, *challenged*… *And those things*, *I think*, *happen when you have limited resources and limited time*, *because you just fall back into doing the everyday”*

*(P3)*

The amount of available *time to generate and develop ideas* negatively affected *adaptability*. Participants highlighted the need for adequate time to reflect on past actions and learn from them. They explained that *time to generate and develop ideas* was essential to support innovative responses to changes within the system. Participants highlighted that working with limited resources it was difficult to find this time as they are busy ‘doing’ instead of ‘thinking’:

*“How can you be innovative if you don*’*t have the resources*?…*You know that having the opportunity and the luxury of being able to think and have new ideas is a luxury these days*.*”*

*(P3)*

Participants recognised the value *adaptability* adds to SiREN, but acknowledged its challenges. They reflected that managing SiREN (e.g. staffing, governance) was challenging when the needs of SiREN and system were in a constant state of change (e.g. new project activities, new research projects). At times they felt their willingness to trial new ways of managing and staffing meant that they did not hold to a particular structure to give it a real chance at success:

*“However*, *I think sometimes we are very flexible in the way we govern*, *and it can be challenging to find the model and hold to it*, *probably*, *for enough time to see it if it works*.*”*

*(P2)*

### Capability

**Fig 3:**
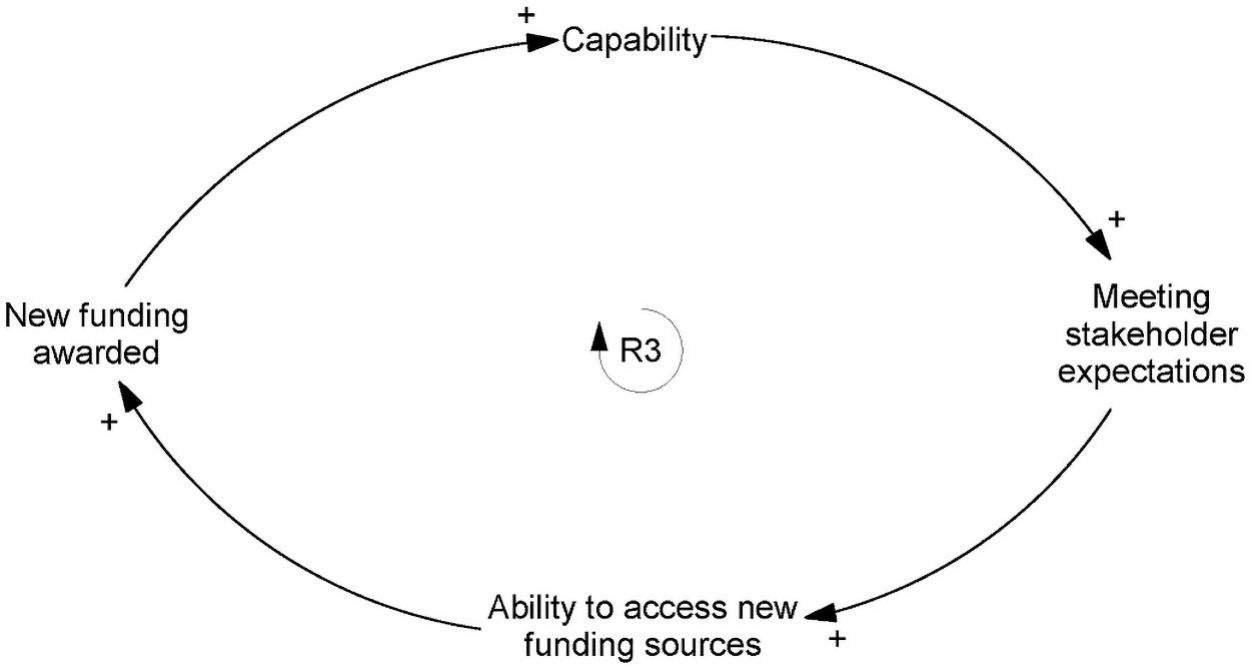
Reinforcing loop two: The relationship between capability and funding.

SiREN demonstrated its *capability* by *meeting stakeholder expectations*, including those of its funders, which enhanced its *ability to access new funding sources*. In turn, greater access to funding sources led to new funding, which enhanced the capability of SiREN, forming a reinforcing loop (Fig 3).

Demonstrating *capability* increased *support from the main funder*. Participants agreed this was a strength, as the funder then linked SiREN to new funding opportunities and promoted it to stakeholders, which increased the *willingness of stakeholders to engage*. Demonstrating capability built trust in SiREN so that when staff identified a new approach, the primary funder supported its trial:

*“But once they (the funder) were happy and could see the growth and the value*, *then I think we were able to change direction*.*”*

*(P4)*

### Credibility

Participants reported SiREN’s *credibility* was enhanced by its quality communications (e.g. electronic communications with its member network). In addition to written communication, interpersonal skills (e.g. responsive, approachable) were fundamental in increasing SiREN’s credibility. SiREN comprised predominantly research-focused staff. Researchers have been criticised for being disconnected from those working in practice [52]. However, a participant reflected that this was not the case with SiREN. They explained this was particularly evident with the project manager who was a “*a skilled broker*.” The project manager and other team members have used their interpersonal skills to build relationships and support the exchange of knowledge across diverse groups, thus building the credibility of SiREN through their *boundary-spanning skills*.

SiREN regularly hosted and presented at events (e.g. conferences, discussion panels) which increased the *visibility of SiREN* and further increased its *credibility*. This process was depicted by a participant:

*“We are constantly at conferences or in conversations*…*We*’*ve also had to kind of build up a bit of a reputation*. *So it*’*s been a process of publications and reports and kind of just starting to kind of lift our profile and be a little bit more visible because previously that never existed*… *And that has been a large volume of work*, *a number of conversations we*’*ve had with consistent messaging across a number of years to be able to have our own spot at the table*.*”*

*(P6)*

SiREN had *associations with credible others*, including high profile researchers, which built credibility by association [53]. This differs from the credibility derived from its *association with a University*, which is attributed to source credibility [54]. A participant described credibility by association:

*“I think that the growth of connections with (national research centres) has been really critical*… *they value what we do*… *they come and speak at our symposiums*. *I think that gives us credibility*.*”*

*(P4)*

Participants also noted that *the longevity of the SiREN increased credibility*. They explained that SiREN has been operating for many years, which built trust in SiREN. Participants observed how the *association with credible others* boosted SiREN’s *credibility* and subsequent ability *to access new sources of funding*.

**Fig 4:**
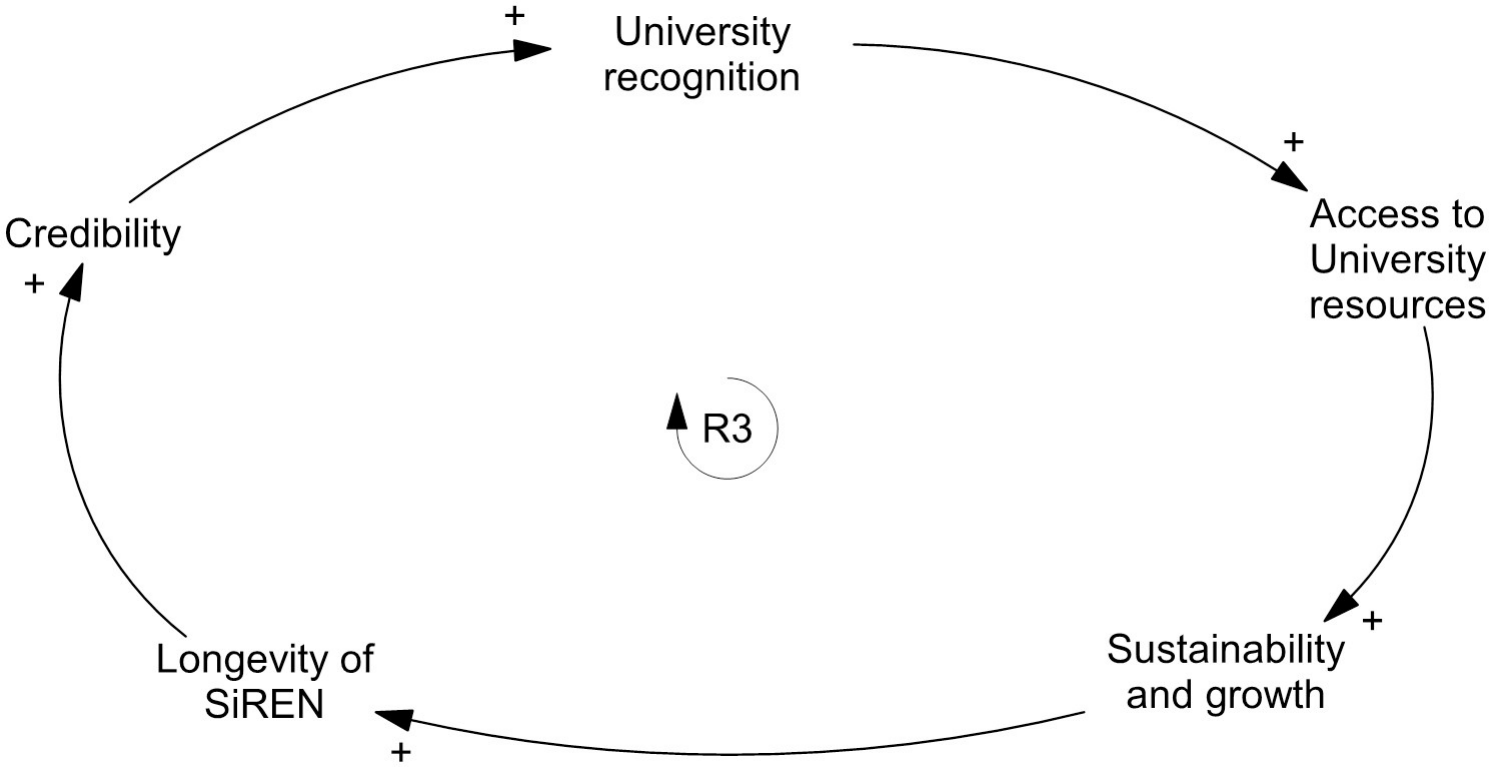
Reinforcing loop three: Credibility and University support.

Participants suggested that the ability of SiREN to generate high quality and impactful research was essential to establishing *credibility* and *capability*. Participants acknowledged the imperative for high-quality research, which to them meant co-produced with stakeholders to meet an identified need. At the same time, they explained their research also needs to demonstrate impact through traditional metrics (e.g. citations) to align the work of SiREN with the University’s objectives and those of being a university-based academic. Aligning SiREN’s aims and activities with the University was considered necessary as it increased *University recognition*, which increased access to *University resources*, such as additional funding to support doctoral students. This had a reinforcing relationship with *sustainability and growth* (Fig 4).

### Sustainability and growth

Key to the *sustainability and growth* of SiREN the amount of *new funding awarded* to SiREN and the strength and diversity of its *stakeholder partnerships*. While SiREN’s partnerships and funding sources had grown since its inception, it remained constrained by inadequate financial resources. *Insufficient funding* limited the ability of SiREN to build capacity and contribute to the generation and translation of research. At times, participants suggested that limited resources affected the level of expertise within the SiREN team by reducing the *attractiveness of the employment terms offered* and *access to professional development opportunities*. Participants identified *staff changes* as a threat to sustainability, as this increased the *time taken to recruit and train staff* and thus reduced *staffing efficiencies*. The effectiveness of SiREN depended on the team’s understanding of, and relationships with, the system. A participant described what this process was like when they first commenced their role:

*“I think that*, *for me*, *it was hard because to do the job well*, *you do need to have time to build up knowledge of the sector*, *and relationships with the people in the sector*. *And they*’*re things that*… *you can*’*t rush them*.*”*

*(P7)*

Situating SiREN within a University had a generally positive impact on *sustainability and growth*. This was because of *access to University resources* such as academic databases, ethical review processes, administrative support, expertise, and *additional human resources* such as postgraduate students to support research projects. Participants noted that being part of the University environment was not without its challenges, mainly related to bureaucracy as significant time was spent on navigating University contracts, administration and ethical processes, which could slow progress.

Participants reported that the most valuable University resource, was the *in-kind time provided by the management team* as this was essential to the sustainability and growth of SiREN. Within this team was a wealth of knowledge on SHBBV, research and evaluation methods, and capacity building approaches that SiREN relied on. The in-kind time that the management team invested in SiREN formed part of their research allocation provided by the University, except for one person who volunteered their time. Therefore, it was generally the University bore the cost of this time. Despite this investment by the University, the time spent on SiREN by the management team consistently exceeded this allocation. The *in-kind time provided by the management team* had diverging effects on sustainability. In some ways it threatened it, while in others it supported it. Some management team members reported that the main funder or University did not recognise the value of their in-kind time. Lack of recognition meant that this time was not costed in SiREN’s budget, masking its real cost. Because this time was not directly financed, pressure from other areas of the management team’s roles within the University diminished the amount of time provided to SiREN. This *workload pressure* occurred when the research allocation provided by the University was reduced or during busy times of semester.

**Fig 5:**
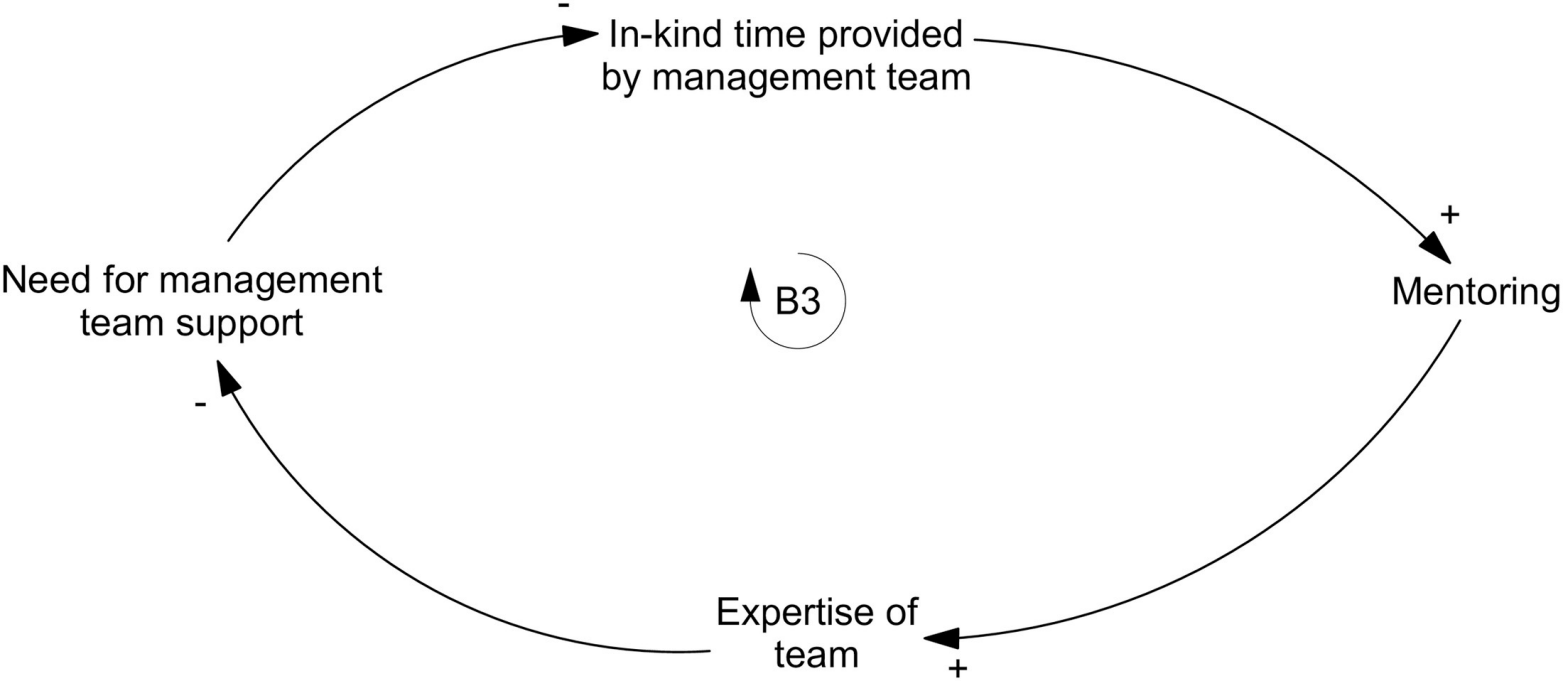
Balancing loop one: Mentoring and the need for management team support.

The management team increased *support available for the team* as they shared their knowledge that strengthened the operation and activities of SiREN and acted as a sounding board for other team members. It also reduced *key person reliance*. Participants explained that SiREN has previously been overly reliant on one staff member to build stakeholder relationships and manage less experienced staff. This dependence threatened sustainability, as there was a risk that relationships and knowledge may be lost if this person left SiREN. An external evaluator identified this risk two years into the operation of SiREN. At which point, this risk was mitigated by shifting some responsibilities (e.g. relationship building, project management) to other management team members. In addition, the management team regularly shared their expertise with less experienced staff. They provided *mentoring*, *sponsorship*, and *supervision of postgraduate students*. *Sponsorship* cultivated a *learning culture* amongst staff, encouraging them to engage in postgraduate study and take up research positions within SiREN that they may not otherwise have pursued. This built the *expertise of the team* over time and reduced the *need for management team support* (Fig 5).

Despite its differing effects on sustainability, the *in-kind time provided by the management team* has offered consistent support to SiREN since its inception. When this research was undertaken, only two management team members had stepped down in eight years, reasons for which were outside SiREN’s control (relocation and changing work roles). This stability was due to the *willingness of the management team to contribute their time*. Willingness was driven by a combination of personal and professional motivations. Firstly, because the management team had a *belief in the ability of SiREN to create change*, there was a good fit between motivation to contribute and SiREN’s aims. Secondly, most management team members have a *personal connection to the HIV epidemic*, which intrinsically motivated them to participate in ongoing prevention and management efforts. Thirdly, rapport and respect between the management team members increased their willingness to invest time. Lastly, SiREN’s outputs, e.g. publications and research grants aligned with their career goals. One management team member reflected that if team members changed, this dynamic might shift, reducing willingness to contribute time. The community-minded nature of the management team also increased their willingness to contribute and led to SiREN exceeding expectations of some funding agreements. Participants reported that this occurred when they were motivated to meet community needs. Participants explained although this added value satisfied funders, it depleted limited resources and could lead to staff burnout.

Participants described tensions and misalignment between University expectations of SiREN, main funder expectations, and the team’s view of SiREN’s role. In the diagram this is reflected under *alignment with funder*’*s needs*. Participants explained that the University endeavoured to be awarded competitive grants and publish in high impact journals. At the same time, the main funder valued tangible *capacity building* outcomes (e.g. number of training sessions held). Participants recognised the importance of both these outputs to SiREN, but explained that they also valued building networks and partnerships to facilitate the co-development and exchange of knowledge. While these value differences have always existed, they have become more pronounced over time and are not unique to SiREN. This is due to growing financial pressures on Australian universities [55] and a lack of incentives provided to staff to engage with communities [56]. Two participants reflected on the conditions when SiREN was initially funded:

*“The climate was right*…*The funding climate*, *the University climate*, *our combined team*’*s climate*. *Everything kind of aligned to go*, *"Yeah*, *you know what*, *we*’*ll put that out if people were happy to fund it*, *support it*.*" So it all just came together*.*”*

*(P1)*

*“If we tried to repeat the origin story of SiREN*, *it would look different now*…*It would look very different*. *I think it would be much harder*.*“*

*(P5)*

## Discussion

This study used systems concepts and methods to explore the internal operation of a research and evaluation capacity building partnership (SiREN). In doing so, it addresses a growing interest in capacity building projects that take a multi-strategic approach and work across the individual, organisational and system level [9, 15] and a need for examples of taking a systems approach to understanding public health programs [19]. The diagram was developed through an iterative and participatory process that involved in-depth interviews, meetings, and workshops with participants. Box 1 provides a summary of key findings relevant to public health practitioners, researchers, or policymakers interested in establishing a similar model.

**Box 1. Critical factors that affected the functioning of a public health research and evaluation capacity building partnership.**

- Adaptability was strengthened by a commitment to a flexible response to meet aims, strong relationships, and knowledge of the system.
- Establishing and building diverse networks and partnerships required an adequate investment of time.
- Consistently demonstrating capability and credibility increased stakeholder willingness to engage.
- Building knowledge of the system required a range of processes (e.g. developing relationships to exchange knowledge, undertaking a needs assessment).
- Cooperation was strengthened by the presence of a shared vision and shared values.
- Expertise and credibility were increased through being located within a university and having access to university resources (e.g. management team)
- Sustainability and growth were enhanced by being cognisant of the different interests and contributions of stakeholders.

Similar to research by Fredericks and colleagues [28], the authors found that the value of causal loop diagrams lies in their ability to bring to the forefront key factors that affect project functioning and strengthen stakeholders’ understanding of a project. Developing the causal loop diagram identified leverage points that can be used to increase SiREN’s success. These new insights were SiREN’s ability to adapt, establish and maintain partnerships, demonstrate capability and credibility and balance different stakeholder interests. This knowledge has since been used to develop a comprehensive evaluation framework (incorporating objectives and indicators) and evaluation tools to support monitoring and evaluation.

Central to SiREN’s ability to achieve its aims has been its capacity to adapt. Adaptation was important as it enabled SiREN to evolve with the needs of the system. It supported the identification of emerging issues that led to the acquisition of new funding sources and increased SiREN’s sustainability. Adaptation was supported by stakeholder partnerships, knowledge of the system and the expertise of the SiREN team. The process of adaptation was challenging within the constraints of the research system, as a swift response to the emerging evidence needs of stakeholders is difficult due to lengthy research processes e.g. preparing funding applications, ethics approvals. Adaptation is a fundamental concept in systems thinking [20, 57]. It has been well explored in the business and management literature for many decades [58–60], yet its use in public health has been limited. This appears to be shifting as public health programs are increasingly viewed as events in systems [29, 30, 61–63]. This change in perspective increases the relevance of how a program interacts with and meets the needs of the system in which it operates. This study found that having a shared vision to work towards and shared values to guide decision making kept SiREN on track while adapting.

SiREN’s strong connections to the system underpinned its ability to adapt. These connections acted as conduits, transferring knowledge between stakeholders and SiREN. SiREN used both formal (e.g. needs assessment, stakeholder meetings) and informal processes (e.g. networking events) to achieve this. As identified in previous research, knowledge sharing was based on trusting relationships [64, 65], and provided SiREN with a valuable understanding of the system which it used to inform the development of its activities (e.g. research project topics, types of training offered). This free flow of knowledge supports adaptation [30, 62]; when this flow is impeded, so too is SiREN’s understanding of the needs of the system. This makes partnership approaches particularly suited to interventions that require adaptation as they can support the exchange of knowledge.

This research found that pre-existing relationships provided a solid starting point for SiREN. Even with this foundational base, significant time was spent establishing and building relationships, particularly as stakeholders entered and exited the system. Consistent with findings from Corbin and colleagues [66], the study determined the accrual of benefits from these relationships takes time. However, once established, the value provided by these relationships was tangible and included quicker responses to emerging issues and greater efficiencies in developing collaborative grant applications. Partnerships also added expertise and credibility to funding applications, thus increasing the likelihood of funding being awarded. These benefits are examples of how capacity building can unlock the potential within a system leading to it being ‘more than the sum of its parts’ [2]. Despite the significant time investment required, findings reinforce the importance of partnerships as key to effectively building research capacity and generating evidence to inform public health decision-making [2, 67].

Like McGill et al. [68], this study found that a systems perspective supported understanding of the level of alignment between SiREN and the interests of system stakeholders. This was evident in SiREN balancing divergent stakeholder needs that can threaten sustainability. SiREN is financially supported by a university, a government department and additional research and evaluation grant funding. These stakeholders have different expectations of SiREN (e.g. research or capacity building outputs). Despite their differing interests, both stakeholders were integral to the sustainability of SiREN. The government funder supported SiREN by encouraging stakeholder engagement and supporting it to adapt as it learned and responded to changes within the system. Being situated within the University was an asset to SiREN as it increased its credibility, the expertise of the team, and access to university resources such as postgraduate students and academic databases. Furthermore, SiREN was often able to find a ‘middle ground’ where it could align its activities across a range of stakeholder needs. For example, co-producing research with stakeholders built research capacity (valued by the main funder) while also achieving research outputs (valued by the University). Those considering developing a similar model should spend time reflecting on the different perspectives of stakeholder partners and how they impact the sustainability of a partnership model.

### Strengths, limitations, and considerations for future studies

Using a systems approach requires defining the boundaries of what is to be included [18]. In doing so, certain elements and perspectives will be excluded. In this study, SiREN partner and service user perspectives were deemed as outside the boundaries. The perspectives of SiREN’s main funder were not included as they declined to participate, citing a conflict of interest. Therefore, the diagram reflects the SiREN teams understanding. Given the nature of boundaries and perspectives, system research will at times be considered insider research. This can be a strength with strategies to reduce bias (e.g. not assuming knowledge, having data and findings reviewed by an outsider) [40]. In this study, including management team members as insider researchers added richness to the diagram as they drew on their deep understanding of SiREN. The authors acknowledge that identifying feedback loops is an important part of developing causal loop diagrams [69]. This study did not report on all feedback loops contained within this diagram as including them all would have complicated the reporting of results. A list of all feedback loops is available from the first author upon request.

The iterative and participatory diagram building process was a strength of this study as it improved the validity of the diagram [28]. Similar to Brennan et al. [45], the authors found involving SiREN team in the model building process added depth to the diagram and developing a shared understanding of SiREN. Participants also felt the diagram crystallised what aspects of SiREN were most valued. However, the iterative nature of refining the model and seeking feedback required a significant investment of time. Traditionally, the development of causal loop diagrams involves collecting data from stakeholders but stops short of including them in the diagram building process [36, 70]. As recognition of the value that participatory approaches bring to the development of causal loop diagrams grows [28, 36, 45, 70], so too should its application. Yet many recent public health studies that have developed a causal loop diagram have not involved stakeholders in the diagram building process [24, 26, 71]. Future studies that utilise causal loop diagrams should weigh the benefits and challenges that collaborative diagram development can bring.

## Conclusion

This study provides insight into critical factors that support the functioning, sustainability and growth of a partnership to build research and evaluation capacity and strengthen evidence-informed decision-making in public health. Key mechanisms for successful functioning were building credibility, capability, strong stakeholder partnerships and knowledge of the system. Adaptability of the partnership within a dynamic system context was an important leverage point to increase its effectiveness. These factors could be applied to partnership models in other public health contexts to facilitate evidence-informed decision-making.

## Supporting information

S1 File Interview schedule and casual loop diagram variables. (DOCX)

S2 File (DOCX)
