# Supplementary material for: Critical factors that affect the functioning of a research and evaluation capacity building partnership: A causal loop diagram
Source: PLoS One. 2022 Jan 13;17(1):e0262125. doi: 10.1371/journal.pone.0262125 (PMC8757999; doi:10.1371/journal.pone.0262125)
Supplement: S1 File — (DOCX) [file pone.0262125.s001.docx]

**S1 Appendix SiREN staff interview schedule.**

1. Could you describe your history of working with SiREN?
2. How well do you think SiREN achieves its aims? Do you think it does what it intends?
3. What factors affect how well SiREN achieves its aims? How do they do this?
4. Is there anything unique to the sector that has influenced SiREN’s ability to achieve its aims?
5. Reflecting on the factors we have just discussed; how do these affect how well you can/could meet the requirements of your role with SiREN? What barriers or enablers do you currently experience? What else might assist you to perform optimally?
6. What factors influence SiREN’s ability to create and respond to new opportunities? E.g. research agenda.
7. Has SiREN’s structure or function changed between its commencement and now, if so, how and why?
8. How do you think SiREN is perceived by the sector?
9. What do you think are the things of value that SiREN provides?
10. Does SiREN need to change to meet the future research and evaluation needs of the sector? If so, in what ways?

**S2 Appendix: Description of causal loop diagram variables.**

Table 1. Description of causal loop diagram variables.

| **Variable** | **Description** |
| --- | --- |
| Ability to access new funding sources | How able SiREN is to identify, apply for, and be awarded new sources of funding. |
| Ability to recruit experienced staff | SiREN aims to recruit staff with the highest level of experience relevant to the role. |
| Access to additional human resources | SiREN has a core team of staff with access to additional human resources as required. This includes new contract staff, and University research students, and volunteers. |
| Access to University resources | Being situated within a University enables SiREN to access University resources including office space, ethics review facilities, students, volunteers, support for the management team to use some of their research allocation to support SiREN’s activities, and funding for higher degree by research students. |
| Adaptability | How SiREN learns from the system and adjusts its processes and activities to respond (e.g., changes in epidemiology). |
| Alignment between SiREN and personal career goals | The degree to which the activities and aims of SiREN align with the career interests and goals of management team members. |
| Alignment with funders needs | How well SiREN meets the expectations of stakeholders that provide financial support (e.g., The University). SiREN has a history of meeting stakeholder expectations. This includes funding conditions which results in successful negotiation for continued funding. |
| Alignment with the University’s strategic plan | Aligning SiREN with the University’s strategic plan ensures a connection between SiREN’s activities and the University achieving its aims. |
| Association with credible others | SiREN has relationships with credible others including high profile researchers, which builds credibility by association (54). |
| Association with the University | Being based within the University gives SiREN source credibility (55). |
| Attractiveness of employment terms | Due to limited funding, SiREN usually employs staff on part-time, fixed term basis. This results in a reduction in the attractiveness of advertised positions to more experienced candidates. |
| Belief in the ability of SiREN to create change | The team believe in the ability of SiREN to indirectly improve health outcomes for people affected by sexual health and blood-borne virus issues. |
| Boundary-spanning skills | How SiREN team demonstrate an understanding of different ways of working, position themselves as approachable, develop relationships and facilitate learning across diverse groups. |
| Capability | The extent to which SiREN can undertake its activities and achieve its aims. |
| Capacity building | SiREN activities that aim to build the research, evaluation, and evidence-informed practice of those working in the system. |
| Commitment to a flexible approach | SiREN is flexible in the kinds of activities it undertakes to achieve its aims. Demonstrated by the team continually seeking to improve its structure and activities. The team are curious about new ways of working and are confident to try new approaches and work in different areas. |
| Community-minded team | The team is motivated to increase research and evaluation capacity within the system. The community-mindedness of the team can be traced back to their personal values, a history of working in stakeholder organisations and a commitment to health promotion values. |
| Conditions of funding agreements are exceeded | On occasion, SiREN has exceeded expected outcomes from a funding agreement. While this demonstrates SiREN’s capability, it is not sustainable as it can deplete limited staff resources. |
| Cooperation between management team members | The team trust in, and understand how to complement, each other’s efforts and abilities. When engaging in dialogue they actively listen and build on each other’s ideas. |
| Credibility | The extent to which SiREN is a trusted and believable source of knowledge. |
| Educational attainment of team | The level of education acquired by the team. |
| Expertise of team | SiREN team have a high level of knowledge and skills across a diverse range of fields (e.g. research, evaluation) as well as subject areas. |
| In-kind time provided by management team | The time that the management team spend on SiREN can form part of their research allocation provided by the University, they may volunteer their time, or a combination of both. |
| Insider knowledge | The team have experience working with and within stakeholder organisations. From this, they have established relationships with stakeholders and an understanding of ‘how things get done.’ |
| Insufficient funding | Inadequate financial resources to support SiREN’s activities and ability to achieve its aims. |
| Key person reliant | The project manager is relied on to maintain stakeholder relationships and oversee SiREN activities. Shared leadership and the in-kind time of the management team can be used to reduce this reliance through sharing the supervision of SiREN activities. |
| Knowledge of the system | SiREN informed is of the system and changes that are occurring within it e.g., changes to epidemiology, evidence needs, evaluation support needs. This knowledge needs to be shared between team members to enable action to be taken. |
| Learning culture | The dynamic culture (norms, values and assumptions) of the team that directs its learning. Within SiREN, staff continuously seek opportunities to learn and apply new skills. |
| Like-minded team | Most members of the team have a shared history of working in stakeholder organisations and the University which has contributed to similarities in how they think. |
| Longevity of SiREN | SiREN has been funded for eight years. This longevity increases the credibility of SiREN. |
| Meeting stakeholder expectations | SiREN has the qualities stakeholders had anticipated or hoped for. This includes meeting informal expectations e.g. responding to a request for support as well as formal expectations e.g. meeting funding agreement terms. |
| Mentoring | The process where the team share their knowledge, skills, and experiences with less experienced staff, students and volunteers. |
| Need for management team support | As the expertise of the team grows there is less need for the in-kind time of the management team. |
| New funding awarded | New financial resources are obtained e.g., research grants. |
| Participation in professional development events | Opportunities to participate in knowledge and skill building events. While mentoring, sponsorship and postgraduate supervision are forms of professional development they are separate variables to illustrate the different effect they have on the functioning of SiREN. |
| Personal connection to HIV epidemic | The management team lived and worked through the HIV epidemic where they saw people they cared about infected and affected by HIV. This experience extended beyond HIV to other sexual health and blood borne virus issues and to marginalised groups. This motivated them to contribute to improving health outcomes within these communities. |
| Quality communication | Communications (e.g. emails, publications, presentations) that are timely, consistent, accurate, and informative build credibility. |
| Rapport and respect between management team | Rapport and respect between management team members increases their willingness to give their time. |
| Research impact and quality | Research impact is about the effect research has on practice or policy and its use to inform further research (70). This is often assessed through journal ranking and article citations. To the team, quality research is co-created, respectful, and shared widely. |
| Researcher profile | Encompasses the research profiles of individuals and the team. Research profile is established through research achievements such as grants, partnerships, publications, and awards. |
| Shared leadership | Developing the vision and activities of SiREN is shared with a steering group and management team. Sharing leadership supports inclusive and transparent decision-making. |
| Shared values | Formed through personal and professional experience predating the inception of SiREN. Shared values are implicit, but without them the cooperative nature of the team would be compromised. They guide the actions of the team. |
| Shared vision | Shared vision is what SiREN needs to accomplish and was shaped by the main funder and stakeholders. Its presence unites the team and stakeholders and supports functioning. |
| Sponsorship | Some management team members sponsor less experienced team members. This process involves advocating and promoting career advancement opportunities for them. |
| Staff changes | Staff leaving and new staff commencing. |
| Staffing efficiencies | Efficiency relates to appropriate levels and utilisation of staff for SiREN to meet its aims. |
| Stakeholder knowledge networks | Stakeholders act as receptors, feeding knowledge about the system (e.g. changes in epidemiology) back to SiREN team which can be used to inform adaptation. These can be formal structures e.g. the steering group or informal e.g. relationships with stakeholders. Stakeholders need to want to share information with the SiREN team, which requires the presence of trusting relationships. |
| Stakeholder partnerships | Partnerships require SiREN and its stakeholders to combine their knowledge, skills, or resources to achieve a shared aim. An example is research co-creation. |
| Supervision of postgraduate students | The supervision of postgraduate students is predominately undertaken by the project manager and the management team. Supervision speaks to maturity of SiREN as there must be capacity within the team to commit time and expertise to guiding the student through to completion. |
| Support available for team | Support comes from the shared leadership structure, specifically the steering group and the management team. These groups give their time in-kind to provide their insight, act as a sounding board, and validate ideas. |
| Support from main funder | The main funder has maintained a clear vision of what it wants SiREN to achieve and supported a flexible approach to how it gets there. It has provided time and support to build relationships. It also encourages stakeholders to seek support from, or partner with, SiREN. |
| Sustainability and growth | The ability of SiREN to acquire and utilise resources to maintain and grow its activities and achieve its aims. Resources include financial, human resources and partnerships. |
| Time away from system | Most management team members have worked with and within stakeholder organisations. For some, this was over a decade ago, and the relevance of their insider knowledge gained has diminished. |
| Time spent meeting University requirements | Working within the University requires SiREN to adhere to its policies and processes e.g., contracts, ethics. This uses up time that could be used on other activities such as building partnerships or pursuing new grant opportunities. |
| Time spent supervising staff | Supervision of staff is predominately undertaken by the project manager and the management team. |
| Time taken to recruit and train staff | New staff need support with navigating University processes, building an understanding of stakeholder organisations, and developing the knowledge and skills required for their role. This is a time intensive process. |
| Time to generate and develop ideas | The team are often busy 'doing' rather than taking the time to identify and develop alternative ways of working. When this occurs it can have a negative impact on adaptation. |
| University recognition | How the University acknowledges the work of SiREN and its contribution to the University aims. The contribution made by SiREN to the University’s aims is limited by its small size making recognition difficult to achieve. |
| Unknown cost of SiREN | The in-kind time provided by the management team is not properly accounted for which means that the real cost of SiREN is not known. |
| Visibility of SiREN | SiREN regularly hosts and presents at events (e.g. conferences, seminars, or forums). This helps build credibility and demonstrate capability. SiREN hosts a biennial symposium which is attended by high profile researchers from the east coast. This visibility increases SiREN’s credibility. |
| Willingness of management team to contribute | The willingness of the management team to contribute their time in-kind is influenced by the dynamics between team members, motivation to improve health outcomes in communities affected by sexual health and blood-borne virus issues, a belief in SiREN’s ability to create change and the alignment between SiREN and their personal career goals. |
| Willingness of stakeholders to engage | How willing stakeholders are to partner with, or request support from, SiREN. |
| Workload pressure | Occurs when time allocated for research in workloads in reduced, during busy times of semester (e.g. marking student assignments), or from pressure from other areas of role. |
